# Right Ventricular Dysfunction and Ventriculo‐Arterial Uncoupling in Heart Failure With Preserved Ejection Fraction: A Phenotype‐Specific Review of Mechanisms, Diagnosis, and Therapeutic Strategies

**DOI:** 10.1002/clc.70489

**Published:** 2026-10-31

**Authors:** Amarelle Chamoun, Talha Farooq, Suhayya Batool, Sadia Tameez‐ud—din, Muhammad Yousaf, Preet Memon, Mohsin Ali, Arun Kumar Maloth, F. N. U. Sawaira, Muhammad Taimur Khan, Mohammad Eisa Ali, Mansoor Khalid, Maleeha Pandit, Kamil Ahmad Kamil

**Affiliations:** ^1^ Faculty of Medical Sciences, Lebanese University Beirut Lebanon; ^2^ Jinnah Sindh Medical University Karachi Pakistan; ^3^ Allama Iqbal Medical College Lahore Pakistan; ^4^ Foundation University Medical College Islamabad Pakistan; ^5^ Dow University of Health Sciences Karachi Pakistan; ^6^ Saidu Medical College Swat Pakistan; ^7^ Kakatiya Medical College Warangal Telangana India; ^8^ Khyber Girls Medical College Peshawar Pakistan; ^9^ Abbottabad International Medical College Abbottabad Pakistan; ^10^ Department of Molecular Medicine University of Pavia Italy; ^11^ Khyber Medical College Peshawar Pakistan; ^12^ Faculty of Medicine Medical University of Plovdiv Plovdiv Bulgaria; ^13^ Chief, Internal Medicine Department Nasrat Bashir Curative Hospital Kandahar Afghanistan

**Keywords:** cardiac magnetic resonance, echocardiography, heart failure with preserved ejection fraction, phenotyping, pulmonary hypertension, right ventricular dysfunction, ventriculo‐arterial coupling

## Abstract

**Background:**

Heart failure with preserved ejection fraction (HFpEF) accounts for nearly half of all global heart failure cases. Historically viewed as a left‐centric diastolic disorder, emerging evidence identifies right ventricular (RV) dysfunction and impaired ventriculo‐arterial (RV‐PA) coupling as central drivers of exercise intolerance, functional decline, and adverse clinical outcomes.

**Objectives:**

This review synthesizes current evidence on the pathophysiological mechanisms, advanced diagnostic modalities, prognostic implications, and phenotype‐specific therapies for RV dysfunction in HFpEF, advocating for a transition to systematic biventricular evaluation.

**Methods:**

A structured, PRISMA‐informed search of PubMed and Scopus (2015–2024) identified 74 eligible studies evaluating RV structure, function, and hemodynamics in HFpEF cohorts.

**Results:**

RV dysfunction is prevalent in 19% to 33% of HFpEF patients and independently predicts all‐cause mortality and heart failure hospitalizations. Key pathophysiological drivers include post‐capillary pulmonary hypertension, impaired RV lusitropy, systemic inflammation, and inter‐ventricular dependence. Non‐invasive markers notably speckle‐tracking echocardiography, 4D‐flow cardiac magnetic resonance (CMR), and the TAPSE/PASP ratio enable early identification of subclinical RV‐PA uncoupling. While traditional heart failure therapies show limited efficacy, emerging options including SGLT2 inhibitors, interatrial shunt devices, GLP‐1 receptor agonists, and targeted pulmonary vasodilators demonstrate potential to preserve RV strain and restore hemodynamics across distinct clinical phenotypes.

**Conclusions:**

RV dysfunction is a major prognostic marker in HFpEF. Routine inclusion of RV‐PA coupling indices in diagnostic and risk‐stratification algorithms is essential to guide precision‐based, phenotype‐guided management and improve clinical trajectories.

## Graphical abstract

Right ventricular dysfunction affects 19%–33% of patients With heart failure With preserved ejection fraction and is associated With exercise intolerance, hospitalization, and mortality. Assessing right ventricular function and its coupling with the pulmonary circulation may improve risk stratification and support management tailored to distinct clinical phenotypes.

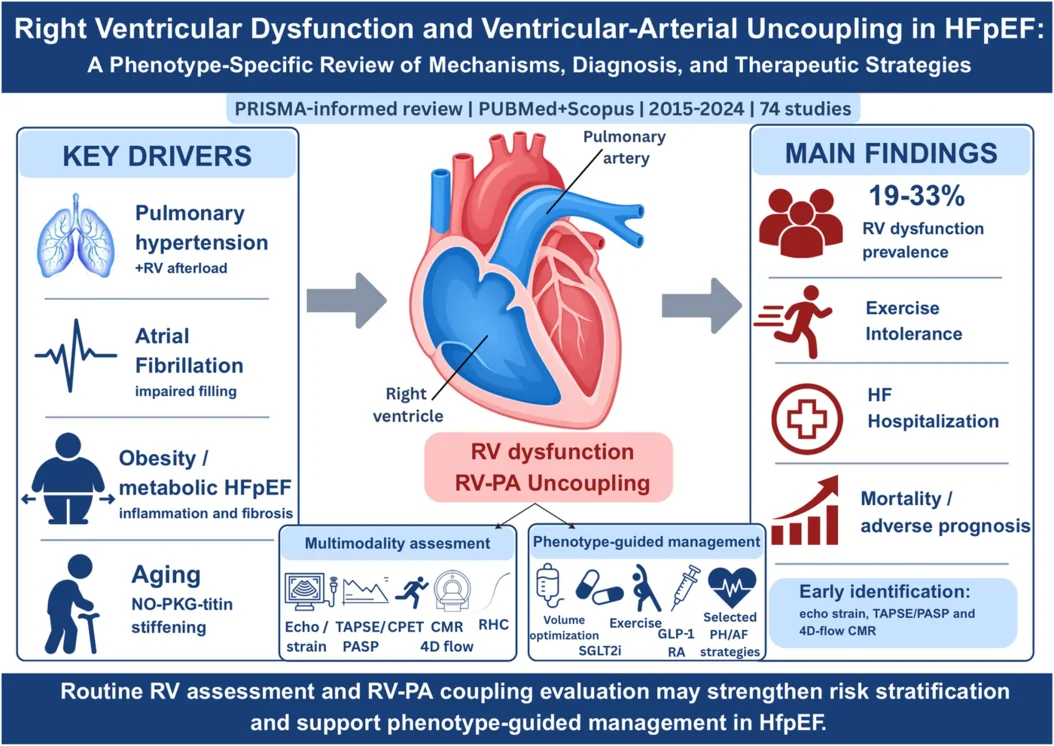

## 1 Introduction

Heart Failure with Preserved Ejection Fraction (HFpEF) represents a complex, increasingly prevalent syndrome, accounting for nearly half of all heart failure cases globally. HFpEF is defined as the presence of heart failure symptoms, such as exertional dyspnea, fatigue, reduced exercise tolerance, peripheral edema, and orthopnea, despite a preserved or relatively normal left ventricular ejection fraction (LVEF ≥ 50%). HFpEF patients are more likely to be elderly, female, or have increased rates of comorbidities such as hypertension, obesity, atrial fibrillation (AF), and diabetes mellitus [1, 2].

HFpEF has been historically classified as primarily resulting from impairments in left ventricular (LV) relaxation and compliance that produce elevated filling pressures and subsequent pulmonary congestion, frequently during periods of exertion [3, 4]. Recent work is pointing towards a more elaborate physiological coordination involving complex interactions with the extracardiac systems and biventricular mechanics, which may include components of the right heart, pulmonary vasculature and peripheral skeletal muscle [5].

For many years, pioneering studies and consensus guidelines have established this diastolic dysfunction explicitly as the main driver of symptoms and adverse clinical outcomes in these patients [3, 6], including recently published landmark ESC and ACC/AHA guidelines, which have emphasized the significance of evaluation of LV filling pressures, myocardial relaxation and hypertrophy in diagnosing and treating HFpEF [7].

Invasive hemodynamic studies and non‐invasive echo indices, such as E/e' ratio and left atrial volume index, are highlighted as key diagnostic elements [8, 9]. This left‐centric view refined diagnostics through specificity and characterization but diminished the relevance of the right ventricle (RV), assigning it a minor role as a passive contributor of left‐sided pathology, only in the form of secondary sequelae. More recently, a growing body of literature has changed the paradigm of RV dysfunction (RVD) as a key determinant of exercise intolerance and poor outcomes in HFpEF.

Advanced imaging and invasive hemodynamics have shown both RV systolic and diastolic abnormalities, including maladaptive RV pulmonary artery coupling, to be common and important independent determinants of outcomes [5, 10, 11, 12].

Importantly, cardiovascular magnetic resonance (CMR) and three‐dimensional echocardiography have shown early RV deformational and deformation reserve impairment, which are associated with decreased functional capacity and elevations in natriuretic peptides [13, 14].

RVD also has important prognostic implications when viewed in the context of pulmonary vascular disease, tricuspid regurgitation, and AF [15, 16, 17]. Modern analyses from large registries, including PARAGON‐HF, have shown RV‐PA coupling indices to be independent predictors of adverse outcomes, including mortality and hospitalization rates, in HFpEF [18, 19].

Considering the growing awareness of the mechanistic and prognostic significance of RVD, this narrative review aims to synthesize existing evidence establishing the contribution of RV systolic and diastolic disorders in HFpEF.

We systematically explore the pathophysiological mechanisms, diagnostic approaches, clinical considerations, and management approaches to RV disease in patients with HFpEF. We review the importance of including RV assessment as part of every HFpEF clinical evaluation and risk stratification algorithm and encourage a transition from unilateral left‐sided HF awareness to a more systematic and biventricular HF evaluation in the ongoing management of patients with HFpEF. We note specific gaps in knowledge and outline proposals for future research to develop treatment for RVD in this complex and heterogeneous patient cohort.

## 2 Methods

This narrative review synthesizes contemporary evidence on right ventricular (RV) dysfunction in HFpEF, focusing on pathophysiology, diagnostic evaluation, prognostic significance, clinical outcomes, and therapeutic strategies. To maximize transparency and minimize selection bias, study selection incorporated a structured, PRISMA‐guided search and dual‐independent screening process. Formal risk‐of‐bias appraisals and meta‐analyses were omitted, consistent with a narrative review design.

### 2.1 Search Strategy

A comprehensive search was executed in PubMed and Scopus for English‐language, peer‐reviewed human studies published between January 1, 2015, and December 31, 2024, using MeSH and free‐text terms (Supporting Information S1: Table 1). We included original research, systematic reviews, meta‐analyses, and case reports evaluating RV function or hemodynamics in HFpEF. Preclinical models, studies focused on HFrEF/HFmrEF or isolated RVD, non‐peer‐reviewed literature, editorials, conference abstracts, and non‐accessible full texts were excluded.

### 2.2 Study Selection Process

Database queries yielded 468 records (PubMed: *n* = 293; Scopus: *n* = 175). Following duplicate removal (*n* = 107) via Rayyan.ai, two reviewers independently screened 361 unique titles/abstracts. Discrepancies were resolved by consensus or a third reviewer. Full‐text evaluation of 171 eligible manuscripts yielded 74 studies for final synthesis. The selection workflow is detailed in Figure 1.

**Figure 1:**
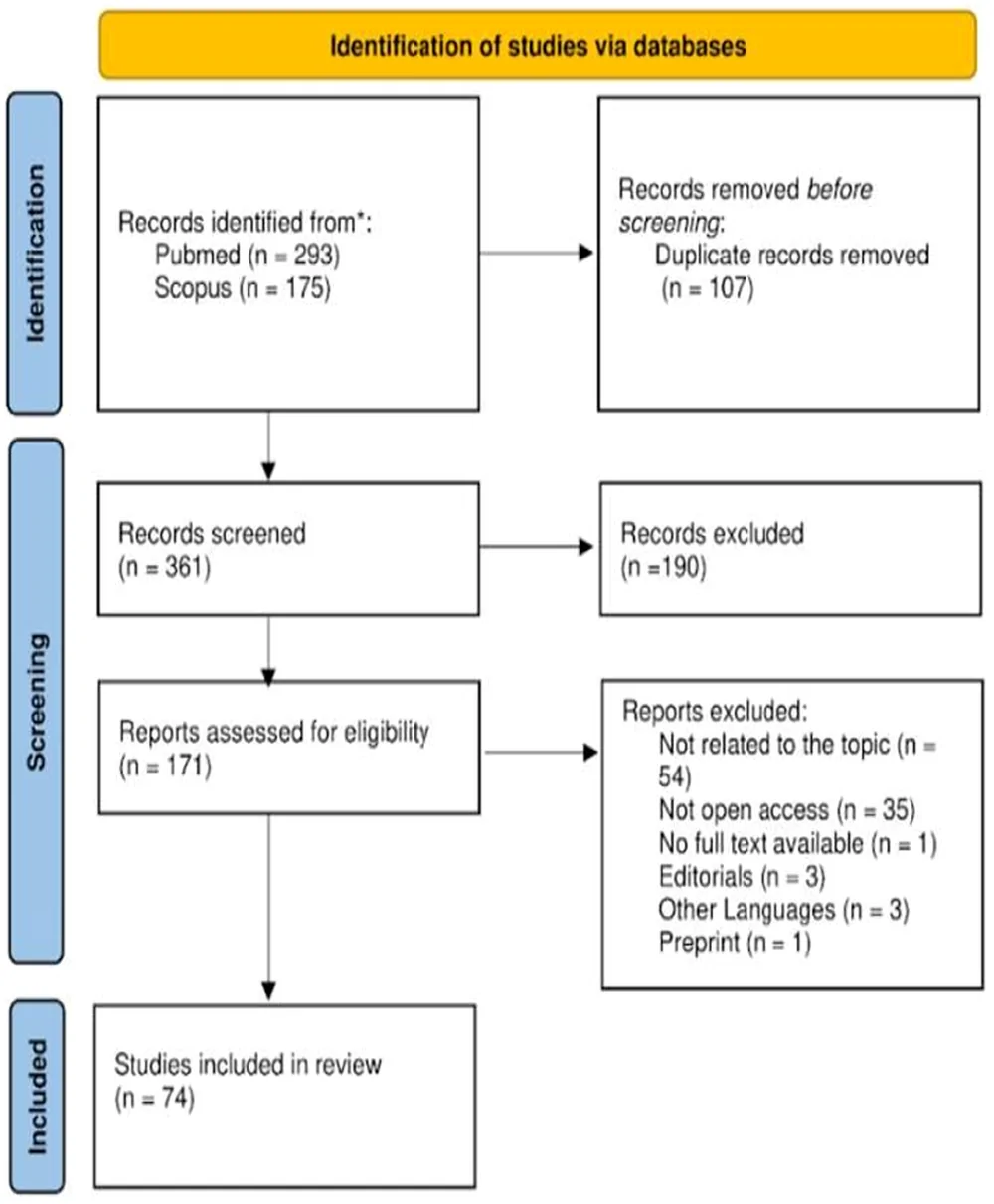
PRISMA flow diagram.

### 2.3 RV Structure and Function

Historically considered less important than the left ventricle, the RV is now recognized as essential for physiological homeostasis, with structural or functional disturbances significantly impacting patient outcomes [20]. The RV is a complex structure with unique anatomical and functional characteristics [20]. Compared to the LV, the RV has roughly one‐sixth the muscle mass but a slightly larger end‐diastolic volume [21].

Anatomically, the RV lies anteriorly and includes anterior, lateral, inferior, and posterior walls, with the latter formed by the interventricular septum (IVS) [22]. In a healthy adult, the RV wall is thin (3–5 mm), highly compliant, and weighs ~25 ± 5 g/m^2^. It is primarily supplied by the right coronary artery, with additional supply from the conus artery and septal branches of the left anterior descending artery [23, 24, 25]. Internally, the RV features a helical arrangement of superficial and deep muscle fibers that facilitate contraction and diastole. Its motion during systole includes longitudinal shortening, radial motion, and anteroposterior shortening [26].

The RV plays a vital role in overall cardiac function and is crucial for patient survival [27, 28]. Functionally, the RV is essential for transferring deoxygenated blood to the pulmonary artery. Contraction begins at the apex and ends at the conus within 20–50 ms, driven primarily by longitudinal shortening [26, 29].

Blood is ejected through a helical flow pattern into the pulmonary artery. RV ejection fraction (RVEF) is a key prognostic marker in diseases such as pulmonary arterial hypertension and correlates well with other imaging modalities like echocardiography and cardiac MRI [30, 31]. For instance, a study found that RVEF predicted the long‐term outcomes in pulmonary arterial hypertension patients equally as transthoracic echocardiography for assessing RV function and served as a reliable alternative to cardiac MRI [30].

The RV demonstrates significant adaptability, with a greater capacity than the LV to handle progressive afterload through contractility and remodeling [21, 32]. Its function is influenced by pressure and volume overload and intrinsic contractility changes, which often coexist in pathology [33]. The RV also has a considerable lusitropic reserve, supporting venous return under high demand via enhanced relaxation and contractility [34, 35].

RV‐arterial coupling, defined by the Emax/Ea ratio, reflects the functional interaction between RV contractility (Emax) and pulmonary arterial load (Ea), with optimal coupling at ratios of 1.5–2 [21, 34, 36]. The RV's contractile reserve allows it to increase output under stress, achieving near‐LV levels of performance during peak exertion [34].

It was recently shown that RV has a notable reserve of stroke volume, and in times of increased O2 demand, RV cardiac output and myocardial energetics are enhanced by ~fourfold from rest to peak effort, approximating observed LV levels [34].

### 2.4 Mechanistic Links Between HFpEF and RVD

HFpEF is a multifactorial disease with a variety of risk factors and comorbidities [37]. Around half of HFpEF patients develop pulmonary hypertension (PH) due to elevated LV filling pressures, worsening prognosis [38]. This increased pressure raises pulmonary venous and systemic vascular resistance, increasing afterload on the RV. However, the RV's limited hypertrophic capacity leads to maladaptive remodeling, dilation, and impaired contractility [39].

A study highlighted how patients with PH‐HFpEF have increased RV mass and interventricular septal angle, while ejection fraction is reduced compared to the LV [40], contributing to symptoms like dizziness, reduced exercise capacity, and leg edema. Accurate diagnostic imaging of the right heart is critical in PH‐HFpEF to assess RV adaptation and systolic dysfunction, both of which are key predictors of patient outcomes [41].

To counter increased pulsatile afterload, the RV engages in ventriculoarterial coupling, a mechanism involving concentric hypertrophy, increased mass‐to‐volume ratio, and contractility to preserve function while minimizing energy expenditure [42]. This is quantified by the Ea/Ees ratio, with a normal value around 1.0. An increased ratio indicates reduced cardiac output and HFpEF progression, ultimately leading to decompensation with reduced stroke volume, chamber dilation, and decreased exercise tolerance [43].

As HFpEF advances, ventriculoarterial uncoupling occurs due to mismatched interaction between the RV and pulmonary arteries. Myocardial fibrosis and stiff ventricular walls impair systolic function and promote systemic inflammation and vascular stiffening [44, 45]. As the hypertrophic RV is unable to preserve cardiac output, free wall hypokinesis occurs, leading to chamber dilatation and an increase in heart rate.

These changes increase arterial load (Ea), impair preload and afterload regulation, and result in decreased Ees/Ea ratios and RV diastolic dysfunction. A figure by Barilli et al. illustrates these mechanisms of coupling and uncoupling in RV adaptation to HfpEF (Figure 2) [45]. Thus, regular monitoring of RV volumes and function is essential to track disease progression

**Figure 2:**
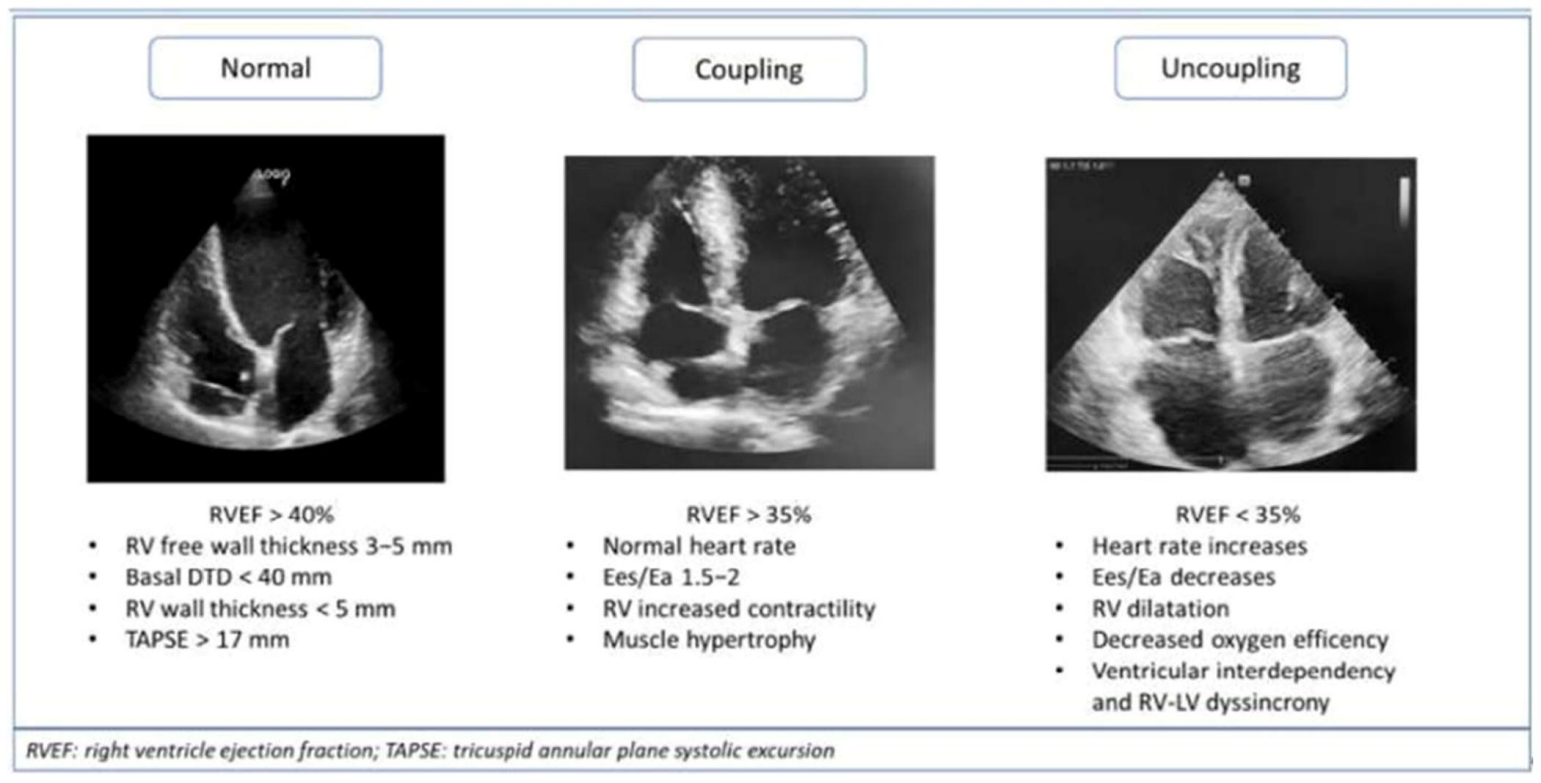
Mechanisms of RV adaption; ventriculo‐arterial coupling and uncoupling (reproduced from Barilli et al. [45]).

Ventriculoarterial uncoupling in HFpEF increases oxygen consumption and leads to ventricular dyssynchrony, the loss of normal timing between RV and LV contractions. Normally, both ventricles function interdependently through synchronized contraction and interventricular septum coordination.

In HFpEF, this interdependence is disrupted as the septum shifts toward the LV, impairing its filling [46]. The study illustrates evidently that in the advanced stages, RV isovolumetric contraction is prolonged while LV has already entered diastole, highlighting progressive chamber discoordination and reduced survival [47]. Figure 3 and Table 1 *summarize the pathophysiologic mechanisms of RVD in HFpEF*.

**Figure 3:**
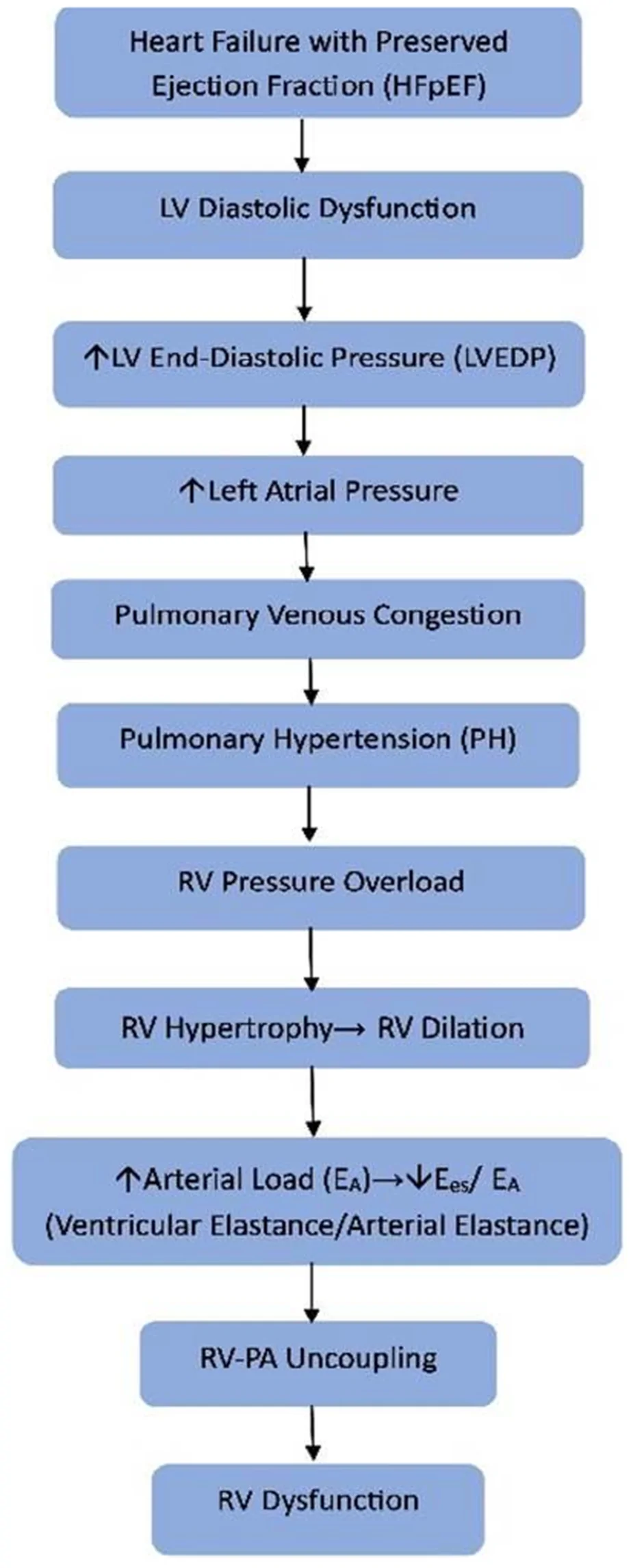
Pathophysiologic mechanisms of RV dysfunction in HFpEF.

**Table 1:** Mechanism, pathophysiology, and clinical relevance of patients with HFpEF.

Mechanism	Pathophysiology	Clinical relevance
Pulmonary hypertension	Elevated left atrial pressure in HFpEF → backward transmission to pulmonary veins → passive pulmonary hypertension → vascular remodeling → ↑ RV afterload	Leads to adaptive hypertrophy, higher right ventricular mass and larger interventricular septal angle and ultimately failure. It causes dizziness and lower limb edema due to reduced ejection fraction and limits exercise tolerance.
Impaired RV‐pulmonary arterial coupling	Mismatch between RV contractility and pulmonary artery load (↑ afterload with insufficient RV inotropy) → free wall hypokinesis occurs → increased heart rate and ventricular decompensation	Decreases right ventricle efficiency leading to increased exertion of heart, decreased stroke volume with ventricular dilatation and lower exercise capacity along with worsening of disease prognosis
Systemic inflammation	Cytokine‐mediated myocardial fibrosis, endothelial activation, and remodeling → increased vascular stiffness → diminished systolic function	Drives pulmonary and cardiac remodelling and can act as a sole factor of HFpEF
Ventricular independence	Increased LV filling pressures or dilation impairs RV filling via interventricular septal shift, especially during diastole	Contributes to RV diastolic dysfunction and exacerbates the symptoms of HFpEF with lower cardiac output and progression of disease with poor survival rate
Endothelial dysfunction	Reduced bioavailability of nitric oxide (NO) → suppression of PKG → hypophosphorylation of titin → development of cardiac hypertrophy and elevated cardiomyocyte tension	Impaired ventricular relaxation and the development of diastolic dysfunction, worsening of RV workload. Promotes pulmonary hypertension with HFpEF
RV dysfunction and comorbidities	Pressure overload and stretching of left atrium → cardiac remodelling → aggravated RV diastolic and systolic dysfunction → intensifies RV impairment	Increases overall cardiac workload and stiffening of chambers, deteriorates life quality and exertion capacity of patients with HFpEF

While earlier literature emphasized increased LV afterload as the main cause of HFpEF, recent evidence points to impaired nitric oxide (NO) signaling as the central mechanism leading to reduce NO bioavailability and microvascular endothelial dysfunction [6].

The reduced NO causes the suppression of protein kinase G (PKG) activity that further causes cardiac hypertrophy and elevated cardiomyocyte resting tension [48]. Thus, these mechanisms are key contributors to HFpEF pathology and should be closely monitored to assess prognosis and survival.

*RVD and comorbidities*: HFpEF generally affects diastolic and systolic functions of RV but comorbidities such as obesity, AF, chronic kidney disease, and diabetes can also majorly influence function. Obesity generally increases the epicardial adipose tissue along with activation of hemodynamic changes such as increased cardiac workload and increased arterial pressure.

It was found in one study that obese patients had more pronounced RV dilatation and more ventricular dysfunction as compared to non‐obese patients [49].

AF can enhance the pulsatile load on the left atrium, decreasing cardiac output due to the irregular length of the cardiac cycle [50]. The vicious cycle between AF and RVD due to HFpEF is well understood. Moreover, diabetes mellitus is a heterogeneous disease, and it also affects patients with heart failure. Although it is associated with left‐sided myocardial remodeling mainly, it has also been shown to affect the systolic and diastolic function of the RV in patients with type 2 diabetes [51].

Several renal factors, including activation of RAAS, anemia, and uremic toxins, along with arterial stiffening and hypertension in chronic kidney disease with HFpEF, can also coexist and cause deterioration of systolic and diastolic function of ventricles of heart [52].

### 2.5 Prevalence and Prognostic Implications

RVD is a common issue in patients with HFpEF, with a prevalence of 19% to 33% [53, 54]. Epidemiological data suggest that it is also associated with a poor prognosis, such as higher hospitalization and mortality rates [54]. One study identified it as the leading cause of death (55.3%) in patients with HFpEF [55]. Another study reported that HFpEF patients with RV systolic dysfunction (RVSD) exhibited significantly greater 1‐year all‐cause mortality than HFpEF patients with normal RV function (41.7% vs. 4.8%, *p* = 0.004) [56].

Other data have also shown that RVD was associated with the primary outcome of death or HF hospitalization, with an adjusted hazard ratio of 3 (*p* < 0.001), suggesting it as an important predictive factor [57].

Moreover, this dysfunction is frequently associated with conditions such as PH, AF, and RV pacing; however, exact relationships are not clearly known [53, 54]. A study reported that RVD patients had lower systolic blood pressure, AF, pulmonary congestion, an increased cardiothoracic ratio, and a higher prevalence of hospitalization than those without RVD [53].

Patients with HFpEF commonly have RVD highly related to an impaired exercise capacity. In a study, RV systolic and diastolic dysfunctions were present in 20%–35% of HFpEF patients, and it contributed significantly to impairment of cardiac output during exercise [17]. In HFpEF, impaired RV function is a key determinant of reduced exercise tolerance through complex interactions involving pulmonary pressures, diastolic function, and skeletal muscle performance. Understanding these mechanisms is essential for developing effective management strategies for patients with HFpEF [1].

Moreover, the prognostic implications of RV systolic and diastolic dysfunction in HFpEF are becoming more widely appreciated as an important predictor of adverse outcomes. A study on heart failure patients showed that the prevalence of RVSD was 30, 34, 32, and 36% based on RV fractional area change (RV FAC), tricuspid annular plane systolic excursion (TAPSE), peak systolic tricuspid annular tissue velocity (Sʹ), and tissue Doppler myocardial performance index (TD MPI) criteria, respectively. Pulsed and tissue Doppler echocardiography revealed 64%, 32%, and 48% prevalence of RV diastolic dysfunction using tricuspid E/A ratio (T E/A), tricuspid deceleration time (TDT), and tricuspid E/E` ratio (T E/E) criteria, respectively [58].

Early identification of RVD in patients with HFpEF is clinically significant due to its association with adverse outcomes. An early diagnosis permits early intervention and may help to manage patients and their prognosis accordingly.

### 2.6 Diagnostic Approaches

#### 2.6.1 Echocardiographic Assessment

Echocardiography is the first‐line diagnostic imaging examination for individuals who have heart failure symptoms and signs [7]. The majority of RVD evidence now comes from echocardiograms [59].

Furthermore, variable diagnostic thresholds (such as TAPSE, fractional area change [FAC], and RVEF) and RV measurement methods influence the reported prevalence of RVD in HFpEF [54]. A summary of the echocardiographic parameters mentioned is presented in Table 2.

**Table 2:** Echocardiographic parameters for assessing RV function in HFpEF.

Parameters	Cut‐off values	Indications	Advantages	Disadvantages	Studies
TAPSE	< 17 mm	Assess RV longitudinal systolic motion	Simple, reproducible	Angle‐dependent, load‐dependent	Rudski et al. [60]
FAC	< 35%	Measures RV Global systolic function	Good global estimate, reflects chamber level function	Depends on image quality, geometric assumptions	Rudski et al. [60]
S'	< 9.5 cm/s	Measures annular systolic velocity	Simple to acquire	Affected by angle and load	Lang et al. [61]
RVEDV (via CMR or 3D Echo)	> normated BSA‐adjusted ranges	Assess RV size and preload	Accurate via CMR	BSA dependent, difficult via 2D echo	Grothues et al. [62]
PASP	> 35−40 mmhg	demonstrates RV afterload and identifies PH	Noninvasive and derived from TR JET	Estimating the RA pressure increases error	Lam et al. [38]
RIMP (pulsed Doppler)	> 0.43 (pulsed Doppler) or > 0.54 (tissue Doppler)	Assess both systolic and diastolic performance	Comprehensive and not dependent on geometry	Affected by arrhythmia and load	Kobalava et al. [16]
TAPSE/PASP	< 0.36 mm/mmHg	Measure of RV‐PA coupling	Non‐invasive, simple, and clinically effective sign of RV contractility. Strong prognostic indicator	Not a direct hemodynamic measurement, just a surrogate	Guazzi et al. [63]

#### 2.6.2 Key Parameters for Evaluating RV Function

TAPSE, FAC, (S‘) lateral tricuspid annulus peak systolic velocity, RVEDV (RV end‐diastolic volume), PASP (pulmonary artery systolic pressure), and MPAP (mean pulmonary artery pressure).

TAPSE, FAC, and S~ are routinely used metrics to assess the RV systolic function [57, 64]. TASPE and FAC have a significant connection and should be considered the key echocardiographic markers for assessing RV function [64]. Regardless of age, gender, or heart rhythm, RIMP has been shown to be a sensitive marker for identifying RV impairment in patients with HFpEF, comprising both systolic and diastolic performance [16].

#### 2.6.3 RV‐PA Coupling and TAPSE/PASP Ratio

Venous return, RV afterload, pericardial compliance, and RV contractility all have an impact on RV performance; nevertheless, RV‐PA coupling is thought to be the most precise and reliable metric of the RV system's functioning. Each parameter only captures one aspect of RV performance.

The RV‐PA coupling, which indicates contractility in the context of afterload, is defined as the ratio of RV end‐systolic elastance (Ees) to effective PA elastance [65]. Although the Ees/Ea ratio is a good indicator of RV‐PA coupling, determining Ees and Ea elastance necessitates real‐time RV pressure and volume measurements in order to generate successive pressure‐volume loops caused by a decrease in venous return via a Valsalva maneuver or stepwise inflation of an inferior vena cava balloon [65]. Rather, because PASP stands for afterload and TAPSE indicates RV contractility, the TAPSE/PASP ratio has been identified as a measure of RV‐PA coupling [65].

Plotting the relationship between RV shortening (TAPSE) and developed force (PASP) is one example of a simple echocardiography‐derived coupling measure that has been proposed and employed in the search for a noninvasive, simple, and clinically effective sign of RV contractility [63]. In contrast to the connection, the ratio of TAPSE/PASP was assumed to give broad information about RV‐PA coupling, with PASP acting as an indirect approximation of Ea and TAPSE as a load‐dependent surrogate of Ees. As a result, it was discovered to have a good predictive value in HFpEF [63].

The average TAPSE/PASP ratio is 1.2 (with a range of 0.8−1.6) mm/mm Hg, which steadily decreases with age [66]. The TAPSE/PASP ratio is a noninvasive method of measuring RV‐pulmonary arterial (PA) coupling in H. It has been shown that RVD, as shown by decreased TAPSE and increased Doppler‐estimated PASP, has a substantial prognostic impact in HF.

#### 2.6.4 Cardiopulmonary Exercise Testing (CPET)

CPET is an evidence‐based tool for assessing exercise capability [67]. A cycle ergometer was used to perform a symptom‐limited cardiopulmonary exercise test, with the goal of obtaining maximal exercise capacity within 8−12 min [13]. The peak VO_2_ was estimated by averaging the previous 30‐s workout. The VE/VCO2 slope was calculated by taking the linear component of minute ventilation (VE) versus carbon dioxide production (VCO2) [68]. In HFpEF, the degree of resting RVD was also inversely proportional to the VE/VCO2 slope [69].

Although both peak V̇O_2_ and V̇E/V̇CO_2_ slope are well‐studied CPET variables in patients with heart failure, the evidence suggests that V̇E/V̇CO_2_ slope is a stronger predictor of prognosis [67, 70].

A higher V̇E/V̇CO_2_ slope is a reliable indication of heart failure prognosis [67, 70].

#### 2.6.5 Cardiac Magnetic Resonance Imaging (CMR)

Previously, imagining the RV's anatomy and function was difficult because of its unfavorable geometry, restricted endocardial surface definition, and atypical contraction pattern. The introduction of laboratory‐controlled hemodynamic examinations and enhanced imaging in clinical practice has enabled extensive RH studies and the collection of the most relevant data [71]. CMR imaging is the gold standard for RV volumetric and functional assessment, as it provides outstanding accuracy and repeatability [62, 72]. CMR's usefulness goes beyond diagnosis clarification to include long‐term disease process monitoring. CMR can assess RV mass exactly, whereas echocardiography cannot. CMR is thus the most accurate imaging choice for determining the volume and function of the right heart [62].

CMR can predict all‐cause mortality and risk stratify patients with HFpEF. RV function and precapillary PH imaging features are the most important predictors of death in people with HFpEF‐associated PH [40]. CMR can also sub‐phenotype myocardial illnesses such as cardiac amyloidosis, hypertensive cardiomyopathy, hypertrophic cardiomyopathy, and cardiac sarcoidosis, all of which cause myocardial stiffness in HFpEF [73].

Also, it is a key non‐invasive imaging technology for diagnosing and tracking patients with PH over time [74, 75], as well as phenotyping patients with HFpEF. The guidelines for the diagnosis and management of PH emphasize the role of CMR in evaluating disease severity and monitoring [76, 77, 78].

#### 2.6.6 4D Flow CMR

Dynamic right heart flow patterns can now be detected and visualized using modern non‐invasive imaging technologies such as 4‐dimensional flow CMR (4D flow CMR), which has thrown new light on RV diastology [79, 80].

A new sequence known as “4D flow CMR” allows for the measurement of vorticity, or the localized spinning motion of blood at specific points, as well as the acquisition and high‐fidelity spatial and temporal visualization of three‐dimensional time‐resolved blood flow and velocities in all directions for each temporal phase of the cardiac cycle [79]. Changes in RV vorticity were detected as markers of RVD in individuals with PAH and/or RV diastolic dysfunction in two pilot studies of RV intracavity diastolic flow. Patients with PAH showed alterations in E‐ and A‐wave vorticity compared to controls, including higher right heart A‐wave velocity, decreased spatially integrated E‐wave velocity, and a lower overall E/A ratio [81]. Another study found that people with RV diastolic dysfunction had reduced early diastole total velocity in the RV chamber [82].

In a study done on 22 patients with LV disease, the analysis of the RV intracavity flow pathline with evidence of decreased kinetic energy at end‐diastole (4D flow CMR) was able to detect early RVD that was not found using standard CMR [83].

#### 2.6.7 RHC

RV end‐diastolic pressure can be assessed non‐invasively by measuring the diameter and collapsibility of the inferior vena cava or directly after right heart catheterization [60].

#### 2.6.8 Pressure Volume Loops

To conduct the standard PV loop analysis methodology, many sets of PV loops at varied preload levels are obtained. RV volumes and pressures must be recorded concurrently (typically using an RV conductance catheter), and varied preload levels can be achieved by partially blocking the inferior vena cava [84, 85] or conducting Valsalva maneuvers [86, 87].

A diastolic elastance curve is another gold standard measure of diastolic performance that the pressure‐volume loop provides. The diastolic stiffness coefficient, β, is found in an equation that represents the diastolic elastance curve, which has curvilinearity and grows with increasing end‐diastolic volume. The severity of PAH is related to the diastolic stiffness of the RV [88].

### 2.7 Prognostic Clinical Subtypes and Phenotypes Related to RV Systolic and Diastolic Dysfunction

HFpEF is a clinically heterogeneous syndrome presenting with a wide spectrum of manifestations. Among these, RVD holds particular significance for disease progression, especially within phenotypes such as PH, obesity/metabolic syndrome, AF, and elderly or frail patients. Each of these subtypes exhibits distinct pathophysiological mechanisms that accelerate both systolic and diastolic RVD and contribute to adverse prognosis.

Distinguishing these phenotypes is not simply a matter of differing hemodynamic loading conditions: the RV cardiomyocyte itself is exposed to phenotype‐specific pathobiological signals inflammatory, metabolic, mechanical, and neurohormonal that determine whether it hypertrophies adaptively or transitions toward maladaptive stiffening and fibrosis. Cellular studies in experimental PH show that both myofibril‐based stiffness, driven by hypophosphorylation of titin, and interstitial fibrosis contribute to RV diastolic stiffening, with myofibril stiffness predominating in early, compensated RVD and fibrosis becoming more prominent as dysfunction progresses [96].

A multimodal study incorporating long‐read RNA sequencing of endomyocardial biopsies from patients with high‐risk PH‐HFpEF identified distinct RV transcriptomic signatures, including differences in titin isoform expression, that distinguished phenogroups with markedly different 1‐year outcomes, providing direct molecular evidence that RV myocyte biology diverges across HFpEF phenotypes [89].

These myocyte‐level adaptations, summarized by phenotype in Figure 4, help explain why RV structural and functional trajectories differ across HFpEF subgroups despite a shared clinical label.

**Figure 4:**
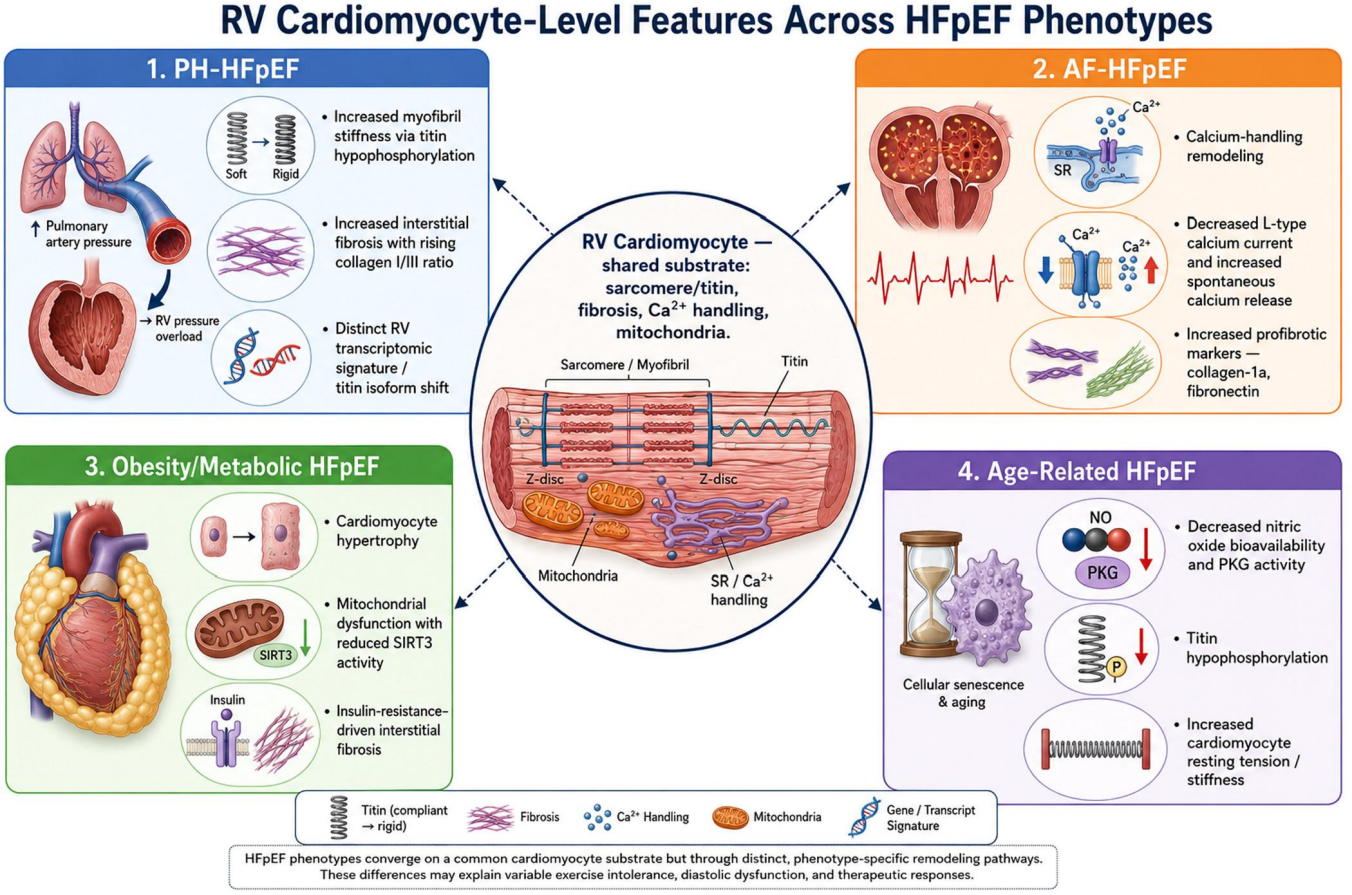
RV Cardiomyocyte‐Level Features Across HFpEF Phenotypes (original schematic synthesizing published experimental and translational data; not reproduced from any single source figure).

#### 2.7.1 AF

Between 30% and 65% of patients with HFpEF experience AF during their disease course [90]. Chronic pressure overload and left atrial dilation induce electrical remodeling and atrial stretch, fostering arrhythmogenesis. The loss of atrial systole (“atrial kick”) directly impairs RV diastolic filling by reducing preload and stroke volume, while beat‐to‐beat variability in cycle length causes mechanical dyssynchrony and fluctuating RV loading conditions. Persistent tachyarrhythmia increases RV wall stress and oxygen demand, promoting progressive systolic impairment.

Mechanistically, the HFpEF–AF phenotype is characterized by elevated fibro‐inflammatory markers, reduced atrial ejection fraction, and left atrial enlargement [91]. Early echocardiographic indicators of RVD in this phenotype include reduced tricuspid annular plane systolic excursion (TAPSE < 17 mm), elevated tricuspid E/E′ ratio > 6, and reduced RV strain by speckle‐tracking. CMR may reveal early strain heterogeneity and reduced RV end‐diastolic volumes.

At the cellular level, the HFpEF–AF phenotype is compounded by calcium‐handling abnormalities. Studies of atrial myocardium from patients with heart failure and AF demonstrate increased expression of profibrotic markers (collagen‐1a, fibronectin, periostin) alongside reduced l‐type calcium current and more frequent spontaneous transient inward currents, changes that promote both contractile impairment and triggered arrhythmic activity [92]. This evidence derives principally from atrial tissue in heart failure with reduced ejection fraction rather than RV myocardium in HFpEF specifically; whether comparable calcium‐handling and fibrotic remodeling extends to the RV in HFpEF‐AF has not been directly tested and represents an important target for future biopsy‐ or biomarker‐based studies.

From a management perspective, rhythm control (via catheter ablation or antiarrhythmics) should be prioritized in AF‐HFpEF patients showing marked RV diastolic impairment, as restoration of coordinated atrial contraction enhances RV filling and overall output [93]. Rate control can be considered in patients unable to tolerate rhythm strategies, although its effect on RV mechanics remains limited.

#### 2.7.2 PH

Elevated LV filling pressures in HFpEF cause retrograde transmission of pressure into the pulmonary vasculature, culminating in post‐capillary PH. Chronic vascular remodeling characterized by medial hypertrophy, endothelial dysfunction, and impaired NO signaling leads to combined pre‐ and post‐capillary PH (Cpc‐PH), substantially increasing RV afterload. Initially, the RV compensates through concentric hypertrophy to preserve contractility; however, persistent pressure overload causes ventriculoarterial uncoupling, dilation, and eventual systolic failure [94, 95].

Microvascular remodeling metrics, such as pulmonary vascular resistance (PVR) > 3 Wood units and diastolic pressure gradient > 7 mmHg, correlate with early RV‐PA uncoupling. Non‐invasive markers like a TAPSE/PASP ratio < 0.36 mm/mm Hg and RV free‐wall strain > –20% are sensitive predictors of early decompensation. CMR further delineates RV Ees and right atrial volume expansion as indicators of maladaptation.

These hemodynamic and imaging findings are supported by cellular data: RV trabecular studies in experimental PH show that myofibril stiffness, driven by titin hypophosphorylation, is the dominant contributor to increased RV diastolic stiffness in early or mild RVD, whereas both myofibril‐ and fibrosis‐mediated stiffness, accompanied by a rising collagen I/III ratio, contribute once RVD becomes severe [96]. This staged progression suggests that interventions restoring titin phosphorylation could theoretically preserve RV compliance before fibrotic remodeling becomes established, although this hypothesis has not yet been tested in HFpEF cohorts.

Therapeutically, patients with Cpc‐PH‐HFpEF may benefit from targeted pulmonary vasodilator therapy such as phosphodiesterase‐5 (PDE‐5) inhibitors or interatrial shunt devices (IASDs), which alleviate RV afterload and restore more efficient RV‐PA coupling [97]. Integrating RV‐PA coupling indices into patient monitoring could guide timely intervention and improve long‐term outcomes.

#### 2.7.3 Obesity

Obesity‐related HFpEF represents a distinct inflammatory‐metabolic phenotype characterized by increased circulating volume, pericardial restraint, and intrathoracic pressure elevation. These factors collectively raise biventricular filling pressures and limit diastolic compliance. Epicardial adipose tissue secretes proinflammatory cytokines (e.g., IL‐6, TNF‐α) and promotes oxidative stress, leading to microvascular rarefaction and RV myocardial fibrosis [94, 98]. Pericardial constraint further exacerbates interventricular interdependence, reducing RV stroke volume and impairing ventriculoarterial coupling.

Early RV functional changes in obesity‐HFpEF include reduced TAPSE, decreased RV longitudinal strain, and increased RV end‐diastolic volumes on CMR, often preceding overt PH.

Direct data on RV cardiomyocyte biology in obesity‐associated HFpEF remain limited. Experimental models of obesity‐related HFpEF demonstrate cardiomyocyte hypertrophy, insulin‐resistance‐dependent interstitial fibrosis, and mitochondrial dysfunction linked to reduced SIRT3 activity, contributing to impaired energy metabolism and increased oxidative stress [99]. These findings come from LV tissue in preclinical models; whether comparable mitochondrial and fibrotic changes occur in the RV myocardium of obese HFpEF patients has not been directly established, but the shared exposure of both ventricles to systemic inflammation and metabolic stress makes this a plausible mechanism warranting dedicated study.

#### 2.7.4 Age

Age‐associated HFpEF primarily exacerbates RV diastolic dysfunction due to myocardial stiffening, vascular aging, and reduced ventricular compliance. Elderly patients with RV involvement demonstrate greater frailty, limited exercise capacity, and higher mortality 91. The interplay between frailty and RV impairment underscores the need for age‐adapted therapeutic strategies emphasizing preload optimization and cautious use of diuretics to preserve RV filling.

At the myocyte level, aging appears to converge on the same NO–PKG–titin axis implicated in HFpEF more broadly: reduced NO bioavailability suppresses PKG activity, leading to titin hypophosphorylation, increased cardiomyocyte resting tension, and hypertrophy [6, 48]. Because this pathway is not RV‐specific, age‐related RV stiffening in HFpEF likely reflects the same systemic endothelial and myocyte‐level processes that drive LV diastolic dysfunction, compounded by the RV's comparatively limited hypertrophic reserve.

HFpEF phenotypes with substantial RV involvement reveal unique mechanistic pathways that drive functional limitation and poor prognosis. Recognition of these mechanisms, coupled with phenotype‐specific diagnostic indices and treatment prioritization, is essential for advancing precision‐based management in HFpEF. Future research should refine diagnostic algorithms and design RV‐focused interventional trials to improve outcomes in this complex patient population.

#### 2.7.5 Advanced HFpEF

RVD is increasingly recognized as an indicator of advanced HFpEF, significantly influencing prognosis and functional capacity. Research suggests that RV involvement in HFpEF is linked to elevated pulmonary pressures, AF, and progressive ventricular remodeling, which contribute to poorer survival outcomes and higher hospitalization rates. A meta‐analysis revealed that individuals with RVD face a 2.28‐fold increased risk of adverse events, with the most pronounced survival differences appearing within the first year of diagnosis [100].

Longitudinal studies further illustrate that progressive RV dilation and tricuspid regurgitation are strongly associated with mortality [101].

Community‐based studies also reinforce that RVD in HFpEF predicts higher cardiovascular mortality and hospital admissions, underscoring its importance in disease progression. These findings highlight the necessity for routine RV function assessment in HFpEF patients to optimize risk stratification and guide targeted therapeutic strategies. The prognostic significance of RVD in HFpEF has been demonstrated in multiple clinical studies (Table 3).

**Table 3:** Summary of key studies on RV dysfunction in HFpEF.

Author + year	Population studied	Study type	RV function measures used	Key prognostic findings
Kanagala et al. [53]	HFpEF (*n* = 135) without PH	Prospective, observational cohort	RVEF (CMR; RVD defined as RVEF < 47%)	RVD independently predicted mortality or HF hospitalization (HR = 3.946, *p* = 0.0001)
Aschauer et al. [55]	HFpEF (*n* = 230); 28.3% with RVD; PH assessed	Prospective, observational cohort	TAPSE (< 16 mm), RV‐FAC (< 35%), RVEF (CMR)	RVD present in 65.8% of non‐survivors; RHF was adjudicated mode of mortality in 55.3% of deaths
Obokata et al. [101]	HFpEF patients (*n* = 271) with/without PH	Prospective, observational cohort	FAC, RV diastolic area, RV GLS, RV FWLS, RVSP	Deterioration in RV function (↓FAC by 10%, ↑RV area by 21%) over 4 years; new RV dysfunction predicted nearly twofold higher mortality (HR 1.89); AF, CAD, obesity were risk factors.
Baratto et al. [13]	HFpEF and PVD patients (*n* = 42)	Prospective, observational cohort	RVEF, RVEDV, RVESV, RV‐PA coupling	60% showed exhausted RV reserve (↓RVEF with exercise), associated with RV dilation, low CO increase, poor exercise capacity (↓peak VO_2_), and intrinsic RV dysfunction; independent of afterload metrics
Garg et al. [40]	HFpEF with PH (*n* = 116)	Prospective cohort	RVEF (CMR), indexed RV mass, interventricular septal angle	RVEF, RV mass, and septal angle were independently predictive of all‐cause mortality. A CMR‐based risk score (RVEF ≤ 49%, RV mass > 17 g/m^2^, septal angle > 139°) stratified mortality.

Abbreviations: CMR = Cardiovascular magnetic resonance, FAC = Functional area change, FWLS = Free wall longitudinal strain, GLS = Global longitudinal strain, HFpEF = Heart failure with preserved ejection fraction, HR = Hazard ratio, PH = Pulmonary hypertension, RVD = Right ventricular dysfunction, RVEF = Right ventricular ejection fraction, RHF = Right heart failure, TAPSE = Tricuspid annular plane systolic excursion.

### 2.8 Management of RVD in HFpER

RVD is increasingly recognized as a major contributor to symptoms, exercise intolerance, and prognosis in HFpEF [12, 59]. Historically, HFpEF was considered a left‐sided heart condition, but substantial evidence has demonstrated the relevance of RV function and RV‐PA coupling to overall cardiovascular performance and outcomes [12, 59].

#### 2.8.1 Hemodynamic Optimization

##### 2.8.1.1 Volume Management

Excess circulating volume increases left atrial pressure and pulmonary venous hypertension, which elevates RV afterload by passive backward transmission through the pulmonary vasculature [7]. Loop diuretics, such as furosemide, act on the Na^+^‐K^+^−2Cl^−^ symporter in the thick ascending limb of the loop of Henle, reducing plasma volume and relieving pulmonary congestion [7]. This leads to symptomatic improvement in dyspnea and edema. However, over‐diuresis may reduce RV preload, compromising stroke volume and cardiac output due to the RV's preload dependence [102].

##### 2.8.1.2 IASDs

IASDs are transcatheter devices that create a controlled left‐to‐right atrial shunt, thereby lowering left atrial pressure and reducing post‐capillary PH [103]. This decreases RV afterload and promotes more favorable RV‐PA coupling. MRI‐based studies show that patients treated with IASDs exhibit stable RV strain over time, while untreated patients display progressive RVD [9].

#### 2.8.2 Pulmonary Vasodilator Therapy

In patients with Cpc‐PH, PVR is elevated, placing significant pressure load on the RV [104]. PDE‐5 inhibitors, such as sildenafil, inhibit cGMP degradation, leading to vasodilation in the pulmonary circulation [104]. This reduces PVR and RV afterload, improving RV‐PA coupling and systolic function [97]. Clinical trials have shown mixed results, but subgroup analyses suggest efficacy in patients with elevated transpulmonary gradients and preserved RV contractile reserve [97].

#### 2.8.3 Sodium‐Glucose Cotransporter 2 (SGLT2) Inhibitors

SGLT2 inhibitors, including dapagliflozin and empagliflozin, have demonstrated benefits in HFpEF populations through several mechanisms. These drugs induce osmotic diuresis, lowering preload and PCWP [105].

They also improve arterial compliance, reduce systemic and pulmonary inflammation, and lower RV wall stress [106]. This contributes to improved RV‐PA coupling and reduced exertional symptoms. The PRESERVED‐HF trial demonstrated that dapagliflozin improved exercise capacity and quality of life in HFpEF patients regardless of diabetic status [107].

#### 2.8.4 Exercise Training

Exercise training enhances skeletal oxygen extraction and improves endothelial function, leading to improved exercise capacity in HFpEF [108]. Peripheral conditioning reduces the cardiac workload needed during activity, indirectly benefiting RV performance. Inspiratory muscle training decreases intrathoracic pressure swings, reducing RV afterload and improving ventilatory efficiency [109]. Exercise also dampens sympathetic overactivation and reduces maladaptive ergoreflex sensitivity, both of which exacerbate RV strain [110].

#### 2.8.5 Phenotype‐Guided Therapies

##### 2.8.5.1 Obesity‐Associated HFpEF

Obesity increases circulating blood volume, intrathoracic pressure, and pericardial restraint, all of which elevate pulmonary pressures and worsen RV function [111]. Weight loss through caloric restriction or pharmacologic therapy reduces these pressures, improving RV filling and exercise tolerance.

##### 2.8.5.2 GLP‐1 Receptor Agonists

GLP‐1 receptor agonists (GLP‐1 RAs), such as semaglutide, support weight loss and exert anti‐inflammatory effects that may favorably remodel cardiac structure [112]. Though specific trials evaluating their impact on RV mechanics are limited, preliminary findings suggest benefit in obese HFpEF subtypes.

#### 2.8.6 AF Management

AF impairs left atrial emptying and raises LA pressures, which transmit backward to the pulmonary vasculature, increasing RV afterload [113].

##### 2.8.6.1 Rhythm Control

Restoring sinus rhythm with catheter ablation improves atrial transport, reduces LA pressures, and enables better LV and RV filling, improving RV output [93]. Ablation may be particularly beneficial in patients with paroxysmal AF and preserved atrial compliance.

##### 2.8.6.2 Rate Control

In patients who are not candidates for rhythm control, pharmacologic rate control (e.g., β‐blockers, calcium channel blockers) reduces tachycardia‐induced inefficiency and prolongs diastole [114]. Although it may offer symptomatic relief, its direct effect on RV function remains uncertain. RVD is a significant and underappreciated determinant of prognosis in HFpEF.

Managing RVD involves careful volume control, improving pulmonary hemodynamics, and addressing comorbidities such as obesity and AF. SGLT2 inhibitors, PDE‐5 inhibitors, IASDs, and structured exercise programs offer varying degrees of benefit. A personalized, phenotype‐based therapeutic approach may yield optimal outcomes in this complex and heterogeneous population. Table 4 provides a summarized comparison of the available therapies.

**Table 4:** Summary of therapeutic approaches for right ventricular dysfunction in HFpEF.

Therapy	HFpEF phenotype (most responsive)	Comparator	Primary endpoints evaluated	Signal consistency^a^	Evidence strength/study design
Loop diuretics (e.g., furosemide)	All congested HFpEF patients	Usual care/no diuretic	Symptom relief, natriuresis, HF‐hospitalization	Consistently improves dyspnea/edema; excess diuresis may reduce RV preload and stroke volume → neutral on mortality	Ponikowski et al. [7], Ter Maaten et al. [102]
Inter‐Atrial shunt device (IASD)	Elevated LA pressure/post‐capillary PH	Sham procedure	LA pressure, RV strain, exercise capacity	MRI studies show stable RV strain over time versus progressive dysfunction in untreated controls	Hasenfuß et al. [103], Backhaus et al. [9]
PDE‐5 inhibitor (sildenafil)	Combined pre‐ and post‐capillary PH (Cpc‐PH) with ↑PVR	Placebo	PVR, RV‐PA coupling, 6‐min walk distance	Mixed overall; subgroup with high transpulmonary gradient shows benefit	Hoendermis et al. [104], Guazzi et al. [97]
SGLT2 inhibitors (dapagliflozin, empagliflozin)	Broad HFpEF (diabetic & non‐diabetic)	Placebo	Peak VO_2_, QoL, RV‐PA coupling	PRESERVED‐HF trial ↑exercise capacity & QoL; meta‐analysis of open‐access studies reports improved RV‐PA coupling and reduced inflammation	Dhingra et al. [105], Packer et al. [106], Nassif et al. [107]
GLP‐1 RA (semaglutide)	Obesity‐related HFpEF	Standard HF therapy	Weight loss, RV strain, inflammatory biomarkers	Pilot data suggest weight‐loss‐driven RV improvement; no definitive outcome trial	Abel et al. [111], Kelly et al. [112]
Structured exercise training	All phenotypes	Usual care	Peak VO_2_, VE/VCO_2_ slope, RV function	Consistently ↑ aerobic capacity and ↓ VE/VCO_2_; indirect RV benefit via afterload reduction	Kitzman et al. [108], Chiappa et al. [109], Olson et al. [110]
Phenotype‐guided interventions (weight loss, rhythm control)	Obesity, AF, CKD sub‐phenotypes	Usual care	RV remodeling, HF‐hospitalization	Small cohort/observational evidence; benefits appear phenotype‐specific‐	Abel et al. [111], Kelly et al. [112], Kotecha et al. [114]

^a^ Signal consistency denotes whether trial results are uniformly positive, mixed, or neutral.

## 3 Research Gaps and Future Directions

To date, research on HFpEF has predominantly focused on LV function, with limited attention to the role of the RV. Identifying existing knowledge gaps and defining future research priorities is essential to advancing pathophysiological insights and optimizing clinical management. Key areas requiring further investigation include the following.

### 3.1 Lack of RV‐Focused Inclusion in HFpEF Trials

Most HFpEF trials focus predominantly on LV function, with insufficient attention given to the RV. This gap limits our understanding of RV's role in the pathophysiology of HFpEF and hinders the development of targeted treatments for RVD in these patients. A study by Von Roeder et al. (2020) highlighted that right atrial‐HFpER coupling is impaired in compensated HFpEF patients, with RV early filling being a strong predictor of exercise capacity. This underscores the importance of incorporating RV function assessments in future HFpEF trials [115].

### 3.2 Need for Standardized RV Diagnostic Criteria

The diagnostic criteria for assessing RV function remain inconsistent across studies. Establishing standardized guidelines for RV assessment could help enhance the accuracy and reproducibility of findings, enabling better comparisons across research and clinical practice. The European Society of Cardiology's guidelines on PH emphasize the need for standardized echocardiographic parameters, such as TAPSE and TAS', to assess RV function and predict PH in HFpEF patients [116].

### 3.3 Prospective Studies on RV‐Targeted Treatments

While there is a growing recognition of RV involvement in HFpEF, prospective studies focusing on treatments specifically targeting the RV are scarce. There is a critical need for such studies to evaluate potential therapeutic strategies aimed at improving RV function and, in turn, patient outcomes in HFpEF.

A study by Huang et al. demonstrated that inhaled iloprost improved myocardial performance during exercise in HFpEF patients by enhancing LV and RV systolic function and reducing PH. This suggests that targeting both ventricles may be beneficial in HFpEF management [117].

### 3.4 Development of Novel Biomarkers and Imaging Tools

Advanced biomarkers and imaging technologies are crucial for assessing RV function in a more precise and non‐invasive manner. The development of such tools could improve both diagnostic capabilities and treatment monitoring, offering valuable insights into RV pathophysiology in HFpEF. Proteomic analyses have identified novel biomarkers associated with HFpEF, such as galectin‐3 and growth differentiation factor‐15, which may reflect myocardial fibrosis and inflammation. These biomarkers could aid in assessing RV involvement and guiding therapeutic decisions [118].

### 3.5 Integrating RV Assessment Into HFpEF Management Algorithms

Current clinical management algorithms for HFpEF often overlook the RV. Incorporating regular RV assessment into these algorithms could provide a more comprehensive approach to managing the disease, potentially improving patient outcomes through better‐targeted therapies. The European Society of Cardiology's guidelines on PH recommend assessing RV function and pulmonary pressures in HFpEF patients to guide treatment strategies and predict outcomes [76].

## 4 Conclusion

RVD has emerged as a crucial, yet historically underappreciated, determinant of prognosis in HFpEF. Although traditional emphasis has been placed on LV dysfunction, emerging research underscores the significant role of RV function in exercise intolerance, functional limitations, and poor outcomes in these patients.

This review highlighted how RVD contributes to HFpEF, its diagnostic challenges, and the impact of RV‐PA coupling on prognosis. It has also stressed the need for routine RV assessment in clinical evaluations and risk stratification, advocating for a more balanced approach to managing both ventricles.

Future research should aim to fill knowledge gaps, especially in RV‐targeted treatments, standard diagnostic criteria, and the development of advanced biomarkers and imaging techniques. Integrating RV function into clinical practice will help improve prognostic accuracy and may lead to targeted therapies that enhance patient outcomes, ultimately improving quality of life and survival rates in HFpEF patients.

## Author Contributions

Mohammad Eisa Ali and Maleeha Pandit contributed to conceptualization and validation. Amarelle Chamoun developed the methodology and contributed to formal analysis and writing the original draft. Talha Farooq, Maleeha Pandit, and Mohammad Eisa Ali contributed to investigation, data curation, and writing the original draft. Suhayya Batool and S.T. contributed to formal analysis and writing the original draft, with S.T. also contributing to review and editing. Muhammad Yousaf and Preet Memon contributed to formal analysis and manuscript review and editing. Mohsin Ali contributed to project administration, coordinating contributions and resolving discrepancies. Arun Kumar Maloth contributed to data curation and formal analysis. F. N. U. Sawaira contributed to investigation and manuscript review and editing, while Muhammad Taimur Khan contributed to data curation and manuscript review and editing. Mansoor Khalid contributed to visualization by creating the graphical abstract. Mohammad Eisa Ali provided supervision, and Mohammad Eisa Ali and Maleeha Pandit also contributed to manuscript review and editing. Kamil Ahmad Kamil served as lead author and led writing, review, and editing. Each author has reviewed and approved the final version of the manuscript.

## Funding

The authors have nothing to report.

## Ethics Statement

The authors have nothing to report.

## Consent

The authors have nothing to report.

## Conflicts of Interest

The authors declare no conflicts of interest.

## Supporting information


Supporting File 1

## Data Availability

The data sets used and/or analyzed during the current study are available from the corresponding author on reasonable request but are available in the public domain and referenced.
