# Supplementary material for: Right Ventricular Dysfunction and Ventriculo‐Arterial Uncoupling in Heart Failure With Preserved Ejection Fraction: A Phenotype‐Specific Review of Mechanisms, Diagnosis, and Therapeutic Strategies
Source: Clin Cardiol. 2026 Oct 31;49(11):e70489. doi: 10.1002/clc.70489 (PMC13723167; doi:10.1002/clc.70489)
Supplement: Supplementary file 1 [file CLC-49-e70489-s001.docx]

**Supplementary Table 1**

**PubMed**

("Ventricular Dysfunction, Right"[Mesh] OR "Right Ventricular Dysfunction" OR "Dysfunction, Right Ventricular" OR "Dysfunctions, Right Ventricular" OR "Right Ventricular Dysfunctions" OR "Ventricular Dysfunctions, Right") AND ("Heart Failure, Diastolic"[Mesh] OR "Diastolic Heart Failure" OR "Diastolic Heart Failures" OR "Heart Failure with Preserved Ejection Fraction" OR "Heart Failure, Preserved Ejection Fraction" OR "Heart Failure with Normal Ejection Fraction" OR "Heart Failure, Normal Ejection Fraction" OR “HFpEF”)

**Scopus**

('right ventricular dysfunction'/exp OR 'right ventricular dysfunction' OR 'ventricular dysfunction, right' OR 'dysfunction, right ventricular' OR 'right ventricular dysfunctions' OR 'ventricular dysfunctions, right') AND ('diastolic heart failure'/exp OR 'diastolic heart failure' OR 'heart failure with preserved ejection fraction' OR 'heart failure with normal ejection fraction' OR HFpEF)

**Supplementary Table 1:** Search strategy for literature review in PubMed and Scopus databases.
